# An uncommon multimodal management approach for low-grade appendiceal mucinous neoplasm with pseudomyxoma peritonei and mucinous borderline ovarian tumor

**DOI:** 10.1097/MS9.0000000000004235

**Published:** 2025-11-04

**Authors:** Dipesh Kumar Singh, Isha Dhakal, Sanskriti Ghimire, Nitesh Pandit, Rajeev Sharma, Soniya Dulal

**Affiliations:** B.P. Koirala Institute of Health Sciences, Dharan, Nepal

**Keywords:** cytoreductive surgery, FOLFIRINOX, HIPEC, low-grade appendiceal neoplasm, mucinous ovarian tumor, pseudomyxoma peritonei

## Abstract

**Introduction and importance:**

Low-grade appendiceal mucinous neoplasm (LAMN) is a rare epithelial tumor characterized by mucin production and potential dissemination into the peritoneal cavity, resulting in pseudomyxoma peritonei (PMP). The coexistence of LAMN with a mucinous borderline ovarian tumor (MBOT) represents a complex and exceedingly rare clinical scenario. Unlike the main introduction, this abstract focuses on the clinical and therapeutic significance rather than epidemiology. There are no standardized treatment protocols for synchronous LAMN with MBOT; hence, individualized management is essential. This report highlights an uncommon multimodal strategy combining cytoreductive surgery (CRS), modified FOLFIRINOX chemotherapy incorporating irinotecan, and planned second-look surgery with hyperthermic intraperitoneal chemotherapy (HIPEC).

**Case presentation:**

A 44-year-old woman presented with abdominal distension. Imaging revealed diffuse mucinous peritoneal deposits, thickened appendix, and multilocular ovarian cyst. Preoperative CA-125 was 142 U/mL, and CA 19-9 was 280 U/mL. CRS including appendectomy, right hemicolectomy, omentectomy, and bilateral salpingo-oophorectomy was performed. Histopathology confirmed LAMN with PMP and MBOT. The Peritoneal Cancer Index at primary surgery was 18. A multidisciplinary team recommended systemic chemotherapy with modified FOLFIRINOX including irinotecan before second-look HIPEC. The patient tolerated the regimen well.

**Clinical discussion:**

This case underscores an emerging role of irinotecan-based chemotherapy as part of a tailored multimodal treatment for low-grade mucinous neoplasms, especially in resource-constrained environments.

**Conclusion:**

The patient remained disease-free at 1-year follow-up, highlighting the potential effectiveness and practicality of irinotecan-based regimens as part of an individualized management strategy for low-grade mucinous neoplasms.

## Introduction

Low-grade appendiceal mucinous neoplasm (LAMN) is an uncommon epithelial tumor originating from the appendix, characterized by mucin-producing neoplastic cells that can disseminate mucin within the peritoneal cavity, leading to pseudomyxoma peritonei (PMP)^[1]^. Although each entity is rare, the synchronous occurrence of LAMN with PMP and a mucinous borderline ovarian tumor (MBOT) represents an exceptionally uncommon clinical presentation that poses diagnostic and therapeutic challenges^[2]^.

**HIGHLIGHTS**

- This case report addresses the management of a rare combination of low-grade appendiceal mucinous neoplasm, pseudomyxoma peritonei (PMP), and mucinous borderline ovarian tumor.
- The use of irinotecan as part of a modified FOLFIRINOX regimen in PMP is novel, bridging a significant knowledge gap.
- It highlights the importance of multidisciplinary collaboration in rare malignancies, particularly in resource-limited settings.
- Recurrence and long-term outcomes emphasize the need for surveillance and tailored follow-up strategies.
- This report underscores the absence of standardized guidelines and calls for more research into systemic chemotherapy for PMP.

The global incidence of LAMN is estimated at 1–2 cases per million annually^[3]^, while reliable epidemiological data from low- and middle-income countries remain limited. In Nepal, underdiagnosis and underreporting are likely due to restricted access to advanced diagnostic imaging and specialized surgical facilities. Accurate diagnosis requires correlation of radiological, surgical, and histopathological findings, as these tumors may clinically mimic advanced ovarian or gastrointestinal malignancies.

Standard management for PMP involves cytoreductive surgery (CRS) combined with hyperthermic intraperitoneal chemotherapy (HIPEC) to remove macroscopic disease and eradicate residual microscopic deposits^[4]^. However, the role of systemic chemotherapy in low-grade mucinous neoplasms remains controversial, as their low proliferative index and limited vascularity may reduce cytotoxic efficacy.

In the absence of standardized guidelines, management must be individualized and guided by multidisciplinary discussion. Recent literature has explored the integration of systemic therapy in selected cases with high tumor burden or synchronous lesions, particularly where delayed or staged HIPEC is planned^[5]^.

This case report describes an uncommon multimodal approach combining CRS, modified FOLFIRINOX chemotherapy incorporating irinotecan, and planned second-look HIPEC in a patient with synchronous LAMN, PMP, and MBOT. The report emphasizes the importance of multidisciplinary decision-making and highlights the potential role of systemic therapy as a bridging strategy in resource-limited settings.

## Methods

Study design: The study is a case report (prospective, unicenter, formal, consecutive, and clinical). The work has been reported in line with the SCARE 2025 criteria^[6]^. The article is compliant with the TITAN guidelines 2025^[7]^.

## Case presentation

A 44-year-old woman from Nepal presented to the Department of Surgery with a gradually progressive abdominal distension and vague lower abdominal discomfort for several months. She denied weight loss, fever, vomiting, or alterations in bowel habits. Her medical and family histories were unremarkable.

On examination, the abdomen was distended with a palpable, non-tender, firm mass in the lower abdomen without signs of acute peritonitis or ascites. Contrast-enhanced computed tomography of the abdomen and pelvis revealed a thickened appendiceal wall, mucinous ascites, and a multilocular cystic lesion involving the right ovary, findings suggestive of a mucinous neoplasm. Laboratory investigations showed elevated serum tumor markers with CA-125 of 142 U/mL and CA 19-9 of 280 U/mL.

The patient subsequently underwent exploratory laparotomy, which demonstrated extensive gelatinous mucinous deposits coating the peritoneal surfaces, with an estimated Peritoneal Cancer Index (PCI) of 18. A thickened appendix and a cystic right ovarian mass were identified.

CRS was performed, including total abdominal hysterectomy, bilateral salpingo-oophorectomy, right hemicolectomy, appendectomy, and infracolic omentectomy. Peritonectomy was deferred during the first operation due to the extensive mucin burden and a planned staged approach with second-look surgery and HIPEC after achieving systemic control. The postoperative period was uneventful. Histopathological examination confirmed an LAMN with PMP composed of acellular mucin, along with a mucinous borderline tumor of the right ovary.

Following discussion in the multidisciplinary tumor board, systemic chemotherapy with a modified FOLFIRINOX regimen was initiated, incorporating irinotecan from the second cycle. The patient completed six cycles of chemotherapy, experiencing only mild fatigue and nausea. Serum CA-125 and CA 19-9 levels normalized after three cycles of therapy.

At the 12-month follow-up, the patient remained clinically stable and radiologically disease free, with normal CA-125 (21 U/mL) and CA 19-9 (30 U/mL) levels, awaiting a planned second-look surgery with HIPEC for complete cytoreduction.

## Discussion

The synchronous presentation of LAMN, PMP, and an MBOT represents an exceptionally rare and diagnostically challenging clinical scenario. Each of these conditions individually is uncommon, and their coexistence poses complex management decisions^[5]^, particularly regarding the role of systemic chemotherapy in low-grade disease.

LAMN is characterized by neoplastic mucin-producing epithelial cells that distend the appendiceal lumen and can disseminate mucin into the peritoneal cavity, leading to PMP^[8]^. The biological behavior of PMP varies according to histopathological grade and the presence of cellular mucin^[9]^. In this patient, the peritoneal deposits were acellular, indicating a low-grade process and generally favorable prognosis. However, the synchronous occurrence of an MBOT and a relatively high PCI suggested a more aggressive biological potential, warranting a broader therapeutic approach.

CRS remains the cornerstone of management in PMP, with the goal of achieving complete macroscopic tumor clearance. The combination of CRS with HIPEC has been shown to improve survival and reduce recurrence rates, with 5-year survival approaching 70–80% in low-grade cases^[10–12]^. However, the optimal integration of systemic chemotherapy in such settings remains controversial.

In this case, a modified FOLFIRINOX regimen incorporating irinotecan was employed as part of a staged, multimodal management plan. The use of systemic chemotherapy in low-grade PMP is unconventional due to the tumor’s low proliferative index and limited vascularity, which reduce drug penetration and cytotoxic efficacy. Nonetheless, the decision to administer chemotherapy in this patient was based on several clinical considerations: the synchronous presence of MBOT, high PCI indicating disease burden, and the need for cytoreduction optimization prior to HIPEC. These features suggested increased neovascularization and potential chemosensitivity.

Irinotecan, a topoisomerase I inhibitor, was chosen for its demonstrated activity in mucinous and gastrointestinal malignancies. Previous pharmacological analyses and small clinical reports have indicated its tolerability and potential benefit in mucinous neoplasms when combined with fluoropyrimidine-based regimens^[12,13]^. The incorporation of irinotecan into modified FOLFIRINOX therefore represents an uncommon but rational approach in selected cases.

The patient tolerated the regimen well, with only mild fatigue and nausea, and demonstrated biochemical response with normalization of tumor markers after three cycles. Serial imaging and tumor marker assessments at 6 and 12 months confirmed disease stability, suggesting effective disease control and supporting the role of systemic chemotherapy as a bridge to HIPEC.

During the planned second-look surgery, the PCI was reduced to 8, and HIPEC using mitomycin-C was successfully performed. The patient experienced no postoperative complications and remained disease-free at 1-year follow-up.

The use of systemic chemotherapy in low-grade mucinous neoplasms remains an area of ongoing investigation. Studies by Govaerts *et al* (2021) and Di Leo *et al* (2020) have described the emerging use of neoadjuvant or bridging chemotherapy in select patients, particularly those with high disease burden or limited initial resectability. This evolving paradigm supports individualized, biology-driven decision-making rather than uniform application of treatment protocols^[8,9]^.

In resource-limited settings such as Nepal, where access to HIPEC facilities is constrained, systemic therapy can serve as a valuable interim measure to achieve disease control and optimize surgical outcomes. This case underscores the importance of a multidisciplinary approach involving surgeons, oncologists, and pathologists to tailor therapy to each patient’s disease biology and resource context.

Overall, this case demonstrates that a staged multimodal approach – incorporating CRS, systemic chemotherapy, and delayed HIPEC – can be both feasible and effective in managing complex synchronous mucinous neoplasms. While further prospective studies are needed, the observed disease stability and favorable short-term outcome highlight the potential role of irinotecan-based regimens in selected low-grade PMP patients.

## Conclusion

This case highlights the successful application of an uncommon multimodal strategy for managing synchronous LAMN, PMP, and an MBOT. The combined use of CRS, systemic chemotherapy with modified FOLFIRINOX incorporating irinotecan, and staged HIPEC demonstrates that individualized treatment based on disease biology and patient condition can achieve meaningful clinical outcomes even in resource-limited environments.

Although the use of irinotecan-based chemotherapy in low-grade PMP is not standard, this case suggests that it may have a role as an emerging or bridging option prior to definitive cytoreduction and HIPEC. The favorable biochemical and radiological response observed underscores its potential in carefully selected patients.

This report emphasizes the importance of multidisciplinary collaboration and adaptive clinical decision-making when established guidelines are lacking. Further research and multicenter data are required to validate systemic therapy strategies and to establish evidence-based protocols for rare synchronous mucinous neoplasms.

## Data Availability

The data that support the findings of this study are available from the corresponding author upon reasonable request.
